# The efficacy and safety of Jin's three-needle therapy vs. placebo acupuncture on anxiety symptoms in patients with post-stroke anxiety: A study protocol for a randomized controlled trial

**DOI:** 10.3389/fpsyt.2022.941566

**Published:** 2022-09-07

**Authors:** Meichen Li, Yuting Wang, Keyi Li, Xiaoyan Xu, Lixing Zhuang

**Affiliations:** ^1^Clinical Medical College of Acupuncture-Moxibustion and Rehabilitation, Guangzhou University of Chinese Medicine, Guangzhou, China; ^2^The First Clinical College, Guangzhou University of Chinese Medicine, Guangzhou, China; ^3^Department of Acupuncture and Moxibustion, The First Affiliated Hospital of Guangzhou University of Traditional Chinese Medicine, Guangzhou, China

**Keywords:** post-stroke anxiety (PSA), Jin's three-needle therapy, sham acupuncture, CORT, ACTH, fMRI, randomized controlled trial, protocol

## Abstract

**Background:**

A large number of clinical RCTs have verified that Jin's three-needle therapy (JTNT) has a great contribution to promoting the function of paralyzed limbs and relieving anxiety disorders for patients with post-stroke anxiety (PSA). However, there is still a lack of sham needle control, and its placebo effect cannot be ruled out. This study firstly verifies the real effectiveness of JTNT. Besides, the changes in serum indexes on the hypothalamic-pituitary-adrenal axis (HPA axis) are observed dynamically by the Enzyme-Linked ImmunoSorbent Assay (ELISA). The activation of different brain regions by JTNT is recorded using resting functional magnetic resonance imaging (rs-fMRI). Therefore, we can provide more practical and powerful evidence-based medical evidence for clinical decisions.

**Method:**

This is a 16 week parallel, single-blind, random, controlled trial, including baseline, 4 weeks of treatment, and 12 weeks of follow-up. A total of 114 participants will be randomly divided into three groups in the proportion of 1:1:1. Participants will receive Jin's three-needle therapy in the active acupuncture group and accept sham needle treatment in the sham acupuncture group. In the waitlist control group, patients will not receive any acupuncture treatment. Outcomes cover three types of indicators, including scale indicators, serum indicators, and imaging indicators. The primary outcome is the change in the performance of anxiety symptoms, which is estimated by the 14-item Hamilton Anxiety Rating Scale (HAMA-14) and the 7-item Generalized Anxiety Disorder scale (GAD-7). Secondary outcomes are physical recovery and daily quality of life, which are evaluated by the National Institute of Health stroke scale (NIHSS) and the Modified Barthel Index Score (MBI Scale). Therefore, the assessment of the scale is carried out at baseline, 2nd, 4th, 8, 12, and 16 weeks. Adrenocorticotropin and cortisol will be quantitatively detected by ELISA at baseline and 4 weeks after treatment. In addition, regional homogeneity analysis (ReHo) will be used to record the activity of brain regions at baseline and 4 weeks after intervention.

**Discussion:**

The study aims to provide high-quality clinical evidence on the effectiveness and safety of JTNT for patients with PSA. In addition, this trial explores a possible mechanism of JTNT for patients with PSA.

**Clinical trial registration:**

Chinese Clinical Trial Registry, identifier [ChiCTR2200058992].

## Introduction

Stroke is the leading cause of permanent disability and death (1). Globally, more than 25 million people are diagnosed with stroke and 6.5 million people die from stroke each year, which aggravates the disease burden (2–4). Despite the existence of global policies and guidelines for stroke implementation, many challenges remain in establishing stroke services (5). In addition to residual physical disability, post-stroke anxiety (PSA) is one of the common neuropsychiatric comorbidities with a high incidence. The reported prevalence of PSA ranges from 20 to 25 percent (6–8). During COVID-19, PSA showed a noticeable peak, which is related significantly to social deprivation and the lack of rehabilitation (9). Compared to non-anxious patients, the patients with PSA are usually in more severe condition at presentation and sustain a long rehabilitation process, resulting in stroke recurrence and death (10–12). It suggests the importance of assessing anxiety and designing effective interventions in chronic stroke survivors. Unfortunately, existing stroke guidelines do not propose the best way to identify and guide the clinical treatment for PSA. Research on PSA is still in its early stages (13). A Cochrane review confirmed that current evidence is insufficient to guide the clinical treatment for PSA (14). Commonly used drugs such as selective serotonin reuptake inhibitors (SSRIs) have poor patient compliance due to their side effects (15). Therefore, more adjuvant therapy methods need to be developed.

Traditional Chinese medicine (TCM) has been used to treat cerebral apoplexy since 3,000 years ago and accumulated abundant and robust evidence (16). Acupuncture, as an important part of TCM, has a solid foundation in China to be applied to improve neurological impairment and complications induced by stroke (17). Jin's three-needle therapy (JTNT), established by Jin Rui, was standardized in one of the National research projects and promoted as the popular acupuncture method in Southern China (18–20). A large number of RCTs have confirmed the efficacy of JTNT in treating mental diseases such as PSA (21, 22). However, there is no placebo-controlled evidence to support it. The real efficacy of JTNT for patients with PSA remains to be proven, and its mechanism has not been fully elucidated.

It is indispensable to identify the cause and pathogenesis of PSA for targeted therapies. According to the available research results, PSA is multifactorial, involving biological and neurophysiological factors. The hypothalamic-pituitary-adrenal (HPA) axis is one of the neuroendocrine systems in the brain that controls the release of glucocorticoids from the adrenocortical glands (23). As well as being a component of the stress response, it also mediates additional downstream pathophysiological changes (24). Hypothalamic hormones stimulate corticotroph cells in the anterior pituitary to secrete adrenocorticotropin (ACTH) (25, 26). ACTH activates the synthesis and secretion of glucocorticoid cortisol (CORT) by the human adrenal cortex (27, 28). As a result of the variety of releasing factors, the HPA axis is expected to respond to stimulation quickly and dynamically. Clinical studies suggest that immediately after stroke onset, a massive release of pro-inflammatory cytokines activates the HPA axis (29, 30). The activated HPA axis promoted a “systemic anti-inflammatory response” that negatively affects function recovery and anxiety generation (31). Besides, anxiety and depression are recognized to be associated with dysregulation of the HPA axis, which accentuates inflammation, consequently increasing the risk of stroke recurrence (32). Dysfunction of the HPA axis is suspected to be one of the main mechanisms connecting stroke with anxiety (33). This study provides preliminary biological evidence for the potential efficacy effect of acupuncture on PSA.

Resting-state functional magnetic resonance imaging (rs-fMRI) is an effective and non-invasive technique to record the activity of the brain in various neuropsychiatric disorders including PSA (34–36). It is revealed that the main regions related to anxiety effects are hippocampus, thalamus, frontal lobe, amygdala, insular cortex, and prefrontal cortex (37–40). The hypothesis of our study is to explore the underlying neural mechanisms of PSA through blood-oxygen-level-dependent effects (41). Regional homogeneity (ReHo) is used to process the fMRI images (42). To evaluate resting-state brain activity effectively, Kendall's coefficient of concordance (KCC) is used to compare a given voxel's time series with its nearest neighbors (43). ReHo theory states that in an area where ReHo is increased, the connections between neurons are strengthened. Conversely, the presence of reduced ReHo indicates a weakening of local connections between neurons. ReHo has shown to be a highly sensitive and reliable method for determining the regional activity level of each voxel in the brain of a single individual (44). Yet, few studies have investigated how acupuncture modulates brain regions in patients with PSA using fMRI. As a result, we use rs-fMRI to explore the possible changes in brain activity and try to find the associations among brain activity, disease activity, and anxiety statuses. This study provides preliminary neurophysiological evidence for the potential efficacy effect of acupuncture on PSA.

In recent years, JTNT has increasingly attracted the attention of clinicians and researchers. However, there is insufficient evidence about the real effects of JTNT on PSA patients. Therefore, we hypothesize that acupuncture will have better clinical efficacy than sham acupuncture in clinical symptoms, anxiety degree, and quality of life. We try to investigate the potential mechanism by enzyme-linked immunosorbent assay (ELISA) and rs-fMRI.

## Methods and analysis

### Ethical standard and study registration

The protocol was registered with the China Clinical Trial Registry (item number: ChiCTR2200058992), and this study protocol has been approved by the Ethics Committee of the First Affiliated Hospital of Guangzhou University of TCM (item number: K2022-02).

### Informed consent

Participants will have sufficient time to decide whether to participate in this trial. Before this trial, patients will have the right to obtain all relevant information about the trial including the benefits and risks, and they will have the right to withdraw from it if needed. As part of the recruitment process, all participants will be required to provide written informed consent before the trial. All participant records will be kept confidential.

### Study design

**Figure 1:**
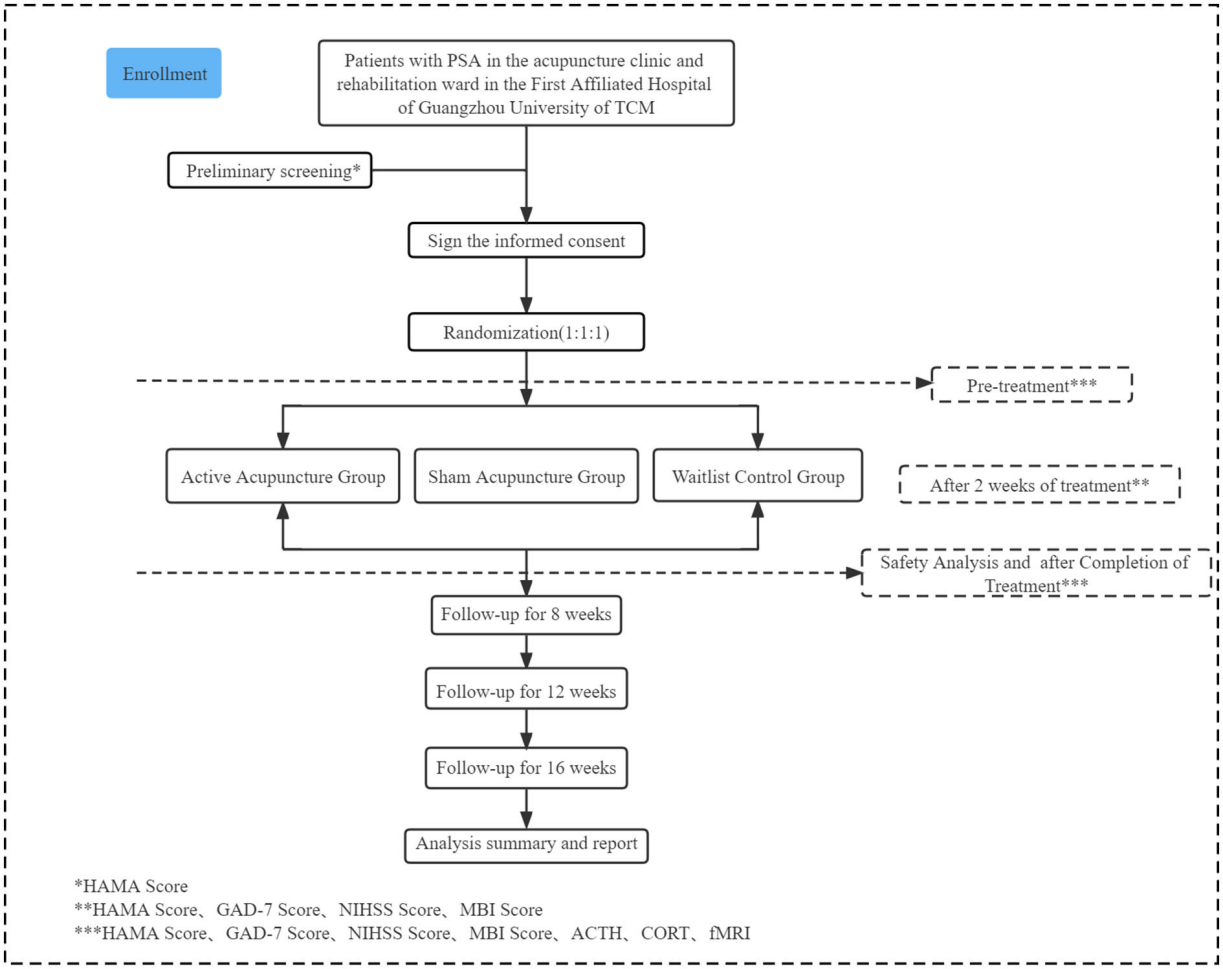
Flow diagram of the study procedure.

A single-center, prospective, randomized controlled trial (RCT) will be conducted at the First Affiliated Hospital of Guangzhou University of TCM. A total of 114 participants with PSA will be recruited and randomly assigned to conventional therapy plus JTNT, conventional therapy plus sham acupuncture, or conventional therapy only. Both JTNT and sham acupuncture are provided five times per week for 4 weeks. Outcomes cover three types of indicators, including scale indicators, serum indicators, and imaging indicators. The primary outcome is the change in the performance of anxiety symptoms, which is estimated by the 14-item Hamilton Anxiety Rating Scale (HAMA-14) and the 7-item Generalized Anxiety Disorder scale (GAD-7). Secondary outcomes are the physical recovery and daily quality of life for patients with PSA, which are evaluated by the National Institute of Health Stroke Scale (NIHSS) and the Modified Barthel Index Scale (MBI). The evaluator will evaluate and analyze the results at six points (baseline, 2nd, 4th, 8, 12, and 16 weeks). ACTH and CORT will be quantitatively detected by ELISA at baseline and 4 weeks of treatment. In addition, ReHo will be used to record the brain regions activity at baseline and 4 weeks after intervention. The flow chart of the trial is illustrated in Figure 1, while Table 1 shows the schedule of measurements.

**Table 1:** Study schedule showing the time points for enrollment and assessment.

	**Study period**							
**Timepoint**	**Enrollment (1 week)**	**Baseline**	**Treatment phase (4 weeks)**			**Follow-up phase (12weeks)**		
	**week 1**	**0 week**	**Week 2**	**Week 4**		**Week 8**	**Week 12**	**Week 16**
**Enrollment**								
Informed consent	×							
Eligibility screen	×							
Medical history	×							
Merger disease	×							
Randomization		×						
Allocation		×						
**Interventions**								
Intervention group		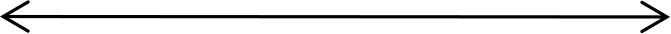						
Control group		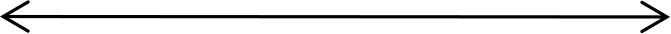						
**Assessments**								
HAMA-14		×	×	×		×	×	×
GAD-7		×	×	×		×	×	×
NIHSS		×	×	×		×	×	×
MBI		×	×	×		×	×	×
CORT		×		×		×	×	×
ACTH		×		×		×	×	×
fMRI		×		×				
**Safety**								
Safety evaluation		×		×				
Adverse events		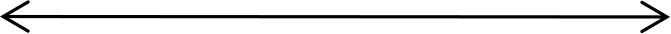				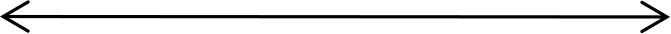		

^×^, required; HAMA, 14-item Hamilton Anxiety Scale; GAD-7, 7-item Generalized Anxiety Disorder-7 Scale; NIHSS, National Institute of Health stroke scale; MBI, the Modified Barthel Index Scale.

### Participant recruitment

Those who occur anxiety more than 1 week after a stroke are diagnosed with PSA according to guidelines customized by The American Psychiatric Association. A target sample of 114 participants will be recruited in the acupuncture outpatient and inpatient systems at the First Affiliated Hospital of Guangzhou University of TCM. The trial will start in May 2022 and run until December 2024. To recruit potential patients, recruitment advertisements will be posted on WeChat, acupuncture outpatient, inpatient systems, and other official platforms. Brief introductions about inclusion criteria, possible benefits for patients, and contact information for the researcher will be provided in the advertisements.

### Inclusion criteria

Participants with the following conditions are included:

(1) The age is between 30 and 75, and gender is not limited.
(2) Diagnosed with stroke (cerebral infarction) within 2 weeks to 3 months and patients who have not had a previous stroke.
(3) Met the diagnostic criteria for “Anxiety disorder due to another medical condition” in the Diagnostic and Statistical Manual of Mental Disorders, Fifth Edition (DSM-V) (45).
(4) The HAMA score ≥14 points, and <29 points.
(5) Clear consciousness, stable vital signs, no cognitive impairment, and ability to cooperate with the scale assessment.
(6) Have not taken anti-anxiety drugs systematically.
(7) An informed consent is signed by patients or their immediate family members.

### Exclusion criteria

Participants with the following conditions are excluded:

(1) Transient ischemic attack, reversible neurological deficit.
(2) Diagnosed with depression, cognitive impairment, schizophrenia, bipolar disorder, substance abuse, or other mental disorders before stroke.
(3) Those who have been confirmed by examination that the neurological deficit is caused by diseases such as brain tumor, brain trauma, cerebral vascular malformation, and brain parasites.
(4) Patients with severe forms of heart disease, liver disease, kidney disease, or tumors.
(5) Contraindication to an MRI examination.
(6) Participating in any other clinical trials.
(7) During pregnancy or lactation.
(8) Patients who suffer from bleeding disorders, coagulation dysfunction, and skin infections that are not suitable for acupuncture.

### Randomization

The randomization sequence will be generated by an independent statistician, who will not participate in other procedures of the trial, using the SAS 9.3 statistical analysis system (SAS Institute Inc., Cary, NC, USA). To guarantee allocation concealment, the generated list of random numbers will be placed into sequentially numbered, opaque, sealed envelopes. A special information manager will be designated to keep information secure. Researchers need to contact the designated information manager to get a random number and group information for each participant included in the study.

### Blinding

**Figure 2:**
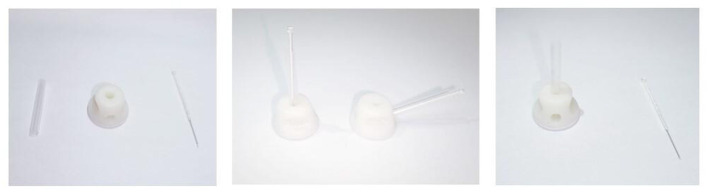
Schematic diagram of the new acupuncture equipment.

Following the information from the envelope, each participant will be randomly assigned in a 1:1:1 ratio to active acupuncture group, the sham acupuncture group or the waitlist group. Acupuncturists, who had received the prepared standard operating procedure guidance for 2 weeks, will be appointed to perform the acupuncture treatment for both groups separately. The blinding acupuncture device has obtained the national utility model patent certificate (patent number: ZL 202121352221.7). It is a validated device that consists of one tube, one needle base with multiple angle openings, one open or sealing adhesive base, and a pointy or flat acupuncture needle. Figure 2 details the blinding device. The customized blinding needles are produced by a manufacturer with relevant qualifications (Guangzhou Suixin Medical Equipment Co., Ltd.). Before our research, patients will be informed that they may have either “less painful acupuncture” (sham needles) or “traditional Chinese acupuncture” (real needles). As the sham acupuncture in this study can create similar pain to acupuncture without penetrating the skin, it can serve as an effective blinding tool. To maximize blinding effect, both groups will be given identical blindfolds during the treatment. Even so, it is impractical to blind the acupuncture operators due to the acupuncture procedure. In an attempt to minimize the subjective influence, statisticians and data managers, as independent three-party to the research, will also be blinded to the group information.

### Interventions and comparison

All groups of patients with PSA will receive routine drugs and rehabilitation treatment during the whole 4-week study, implemented by clinicians and rehabilitation therapists with more than 5 years of clinical experience. All the researchers in this study had received the prepared standard operating procedure (SOP) guidance for 2weeks before the trial. According to the Guidelines for the Prevention and Treatment of Cerebrovascular Diseases in China (46), basic therapeutic drugs with nutritional support, neuroprotection, and secondary prevention drugs will be provided. Additionally, rehabilitation exercises targeting different functions of the subject will be implemented. However, Chinese herbal medicine will be prohibited during the trial. The active and sham acupuncture groups will receive acupuncture treatment five times per week for 4 weeks. The waitlist group will not receive any acupuncture treatment. The specific operations are as follows:

### Active acupuncture group

**Figure 3:**
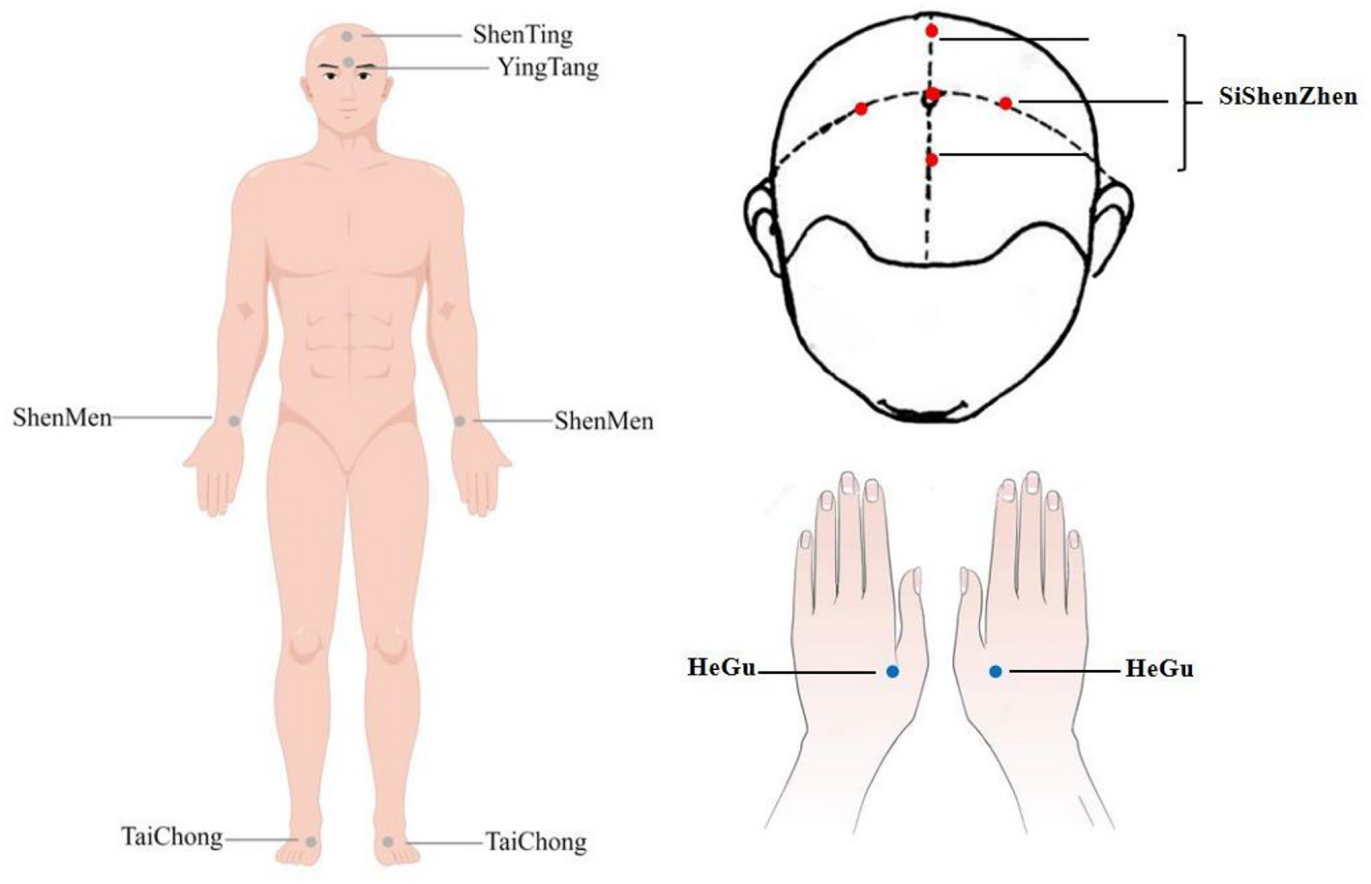
Location range of acupuncture points.

Jin's three-needle therapy is performed by professional acupuncturists. After the acupoint is positioned precisely and the skin to be punctured is disinfected, the needle will be inserted quickly. The location of acupoints is based on the guidelines issued by the World Health Organization (WHO) (47). The subject is asked to take a supine position and wear an eye patch after fully exposing the site to be punctured. Before the intervention, the acupuncturists assemble the tube into the needle base and put the open adhesive base stacking on the target acupoint after skin disinfection with an iodophor cotton swab. The pointy acupuncture needle is passed through the tube into the skin of the subject. Figure 3 shows the acupuncture points, and Table 2 details the locations. Acupoints will be stimulated manually with the depth of insertion varies from 0.5 to 1.5 cun until operating the sense of “De Qi” (48). The reactions of “De Qi” make patients feel soreness, distension, or heaviness, which are vital for acupuncture to react to different nerve conduction (49, 50). After “De Qi” happens, the needles are kept in the acupoints for 30 min without electricity applied. After the needle is removed, the needle hole will be pressed with a sterile dry cotton swab for a while. It must be ensured that the patient is not allowed to remove the eye patch from the start of the acupuncture to the end of the needle withdrawal procedure.

**Table 2:** Specific acupoint location and angle requirements.

**Acupoints**	**Location**	**Insert angle**	**Insert depth**
BaiHui (GV20)	on the head, 5 cun directly above the midpoint of the anterior hairline, or at the midpoint of the line connecting the apexes of the two auricles.		
SiShenZhen	On the head, a total of 4 acupoints are distributed in the front, back, left and right sides of the Baihui acupoint, with 1.5 cun each above BaiHui.	15°	0.5 cun
ShenTing (GV24)	on the head, 0.5 cun directly above the midpoint of the anterior hairline.	15°	0.5 cun
YinTang (GV29)	On the forehead, at the midpoint between the two medial ends of the eyebrow.	15°	0.5 cun
ShenMen (HT7) (bilateral)	On the wrist, in the ulnar depression of the transverse wrist crease, the radial border of the flexor carpi ulnaris tendon.	90°	0.5 cun
SanYinJiao (SP6) (bilateral)	On the inner side of the calf, 3 cun above the tip of the medial malleolus, along the medial border of the tibia.	90°	1.5 cun
HeGu (LI4) (bilateral)	On the dorsum of the hand, approximately on the radial side of the second metacarpal bone, between the first and second metacarpal bones of the hand.	90°	0.5 cun
TaiChong (LR3) (bilateral)	On the dorsal side of the foot, in the subdigital depression to the first metatarsal space.	90°	0.5 cun

1 cun is equal to the width of the interphalangeal joint on the middle finger.

### Sham acupuncture group

Before the intervention, the acupuncturists assemble the tube into the needle hub and put the sealing adhesive base stacking on the target acupoint after skin disinfection. However, the flat acupuncture needle is passed through the tube into the adhesive sealing base, and the peak of the flat needle can touch the surface skin of the subject to create similar pain as real acupuncture without penetrating the skin. Therefore, it can serve as an effective blinding tool. In both groups, the number of needles inserted, needle retention time, and treatment frequency are the same.

### Waitlist control group

During the first 4 weeks, participants in the waitlist group will not receive any acupuncture intervention but will be given compensatory needle therapy after follow-up phase finished.

### Sample size

The sample size was calculated by the primary outcome. According to the results of preliminary pre-experiment, the Mean ± SD of HAMA score in patients who received conventional therapy plus JTNT was 14.25 ± 7.10, while it was 19.46 ± 4.80 in patients who received conventional therapy plus sham acupuncture. Statistical power is set at 0.90, alpha is set at 0.05, and two-sided tests are performed. After calculations performed by PASS 15.0 (NCSS LLC. Kaysville, Utah, USA) software, 30 people are estimated for each group. The estimated loss to follow-up rate is 20%, so each group will eventually need to include 38 people. The formula for sample size calculation is as follow. Therefore, to verify the placebo effect of sham acupuncture, a waiting group was recruited. The number of participants in the waitlist control group is equal as the sham acupuncture group. Finally, a total of 114 participants will be randomly divided into three groups in the proportion of 1:1:1.


(1) $$\begin{array}{l} N=\frac{{\left(Z_{\alpha }+Z_{\beta }\right)}^{2}*2\sigma^{2}}{\delta^{2}} \end{array}$$

### Outcome measures

Outcomes cover three types of indicators, including scale indicators, serum indicators, and imaging indicators. The assessment of the scale is carried out at baseline, 2nd, 4th, 8, 12, and 16 weeks. CORT and ACTH will be quantitatively detected by ELISA at baseline and 4 weeks of treatment. In addition, ReHo will be used to record the activity of the brain at the baseline and 4 weeks after intervention. All evaluations are conducted by researchers who are blinded to the treatment allocation.

### The primary outcome

The primary outcome is the change in the performance of anxiety symptoms, which is estimated by the 14-item Hamilton Anxiety Rating Scale (HAMA-14) and the 7-item Generalized Anxiety Disorder scale (GAD-7).

#### Hamilton anxiety rating scale

The severity of anxiety is often determined by psychological scales. The Hamilton Anxiety Scale (HAMA) is one of the first rating scales to measure the severity of perceived anxiety symptoms. It is considered one of the most widely used rating scales and has been translated into Cantonese (51). A structured interview guide contains a 14-item Hamilton Anxiety Rating Scale of general anxiety, which is rated on a 5-point scale and ranged from 0 (none) to 4 (very severe) by the interviewer.

#### Generalized anxiety disorder scale

Several different types of anxiety disorders have been found in PSA. GAD seems to be the most common type (11). The 7-item Generalized Anxiety Disorder Scale (GAD-7) is commonly used to monitor anxiety symptoms. The reliability and validity of GAD-7 have been well-documented (52). A diagnostic meta-analysis in East Asian samples concluded that given the brevity, sensitivity, and specificity of the questionnaire reported, GAD-7 can be well-utilized to identify people with GAD (53).

### The secondly outcome

The secondary outcomes are the physical recovery and daily quality of life for patients with PSA, which are evaluated by the National Institute of Health stroke scale (NIHSS) and the Modified Barthel Index Scale (MBI). Besides, secondary outcomes will be obtained using ELISA and fMRI scanning. ELISA will be used for quantitative analysis of the content of CORT and ACTH in serum.

#### National institute of health stroke scale

Stroke severity is measured using the National Institute of Health Stroke Scale (NIHSS) (54). There is a 15-item neurologic examination in the NIHSS that is used to assess stroke symptoms. The items are graded from 0 to 42 on an ordinal scale, with higher scores indicating greater severity.

#### Modified barthel index scale

The Modified Barthel Index (MBI) contains ten basic aspects assessing functional independence related to self-care and mobility (55). Each item is divided into five levels, and the level represents different degrees of independence. The lowest level is 1 and the highest is 5, while the higher the level, the greater the independence. The normal score is 100.

#### Enzyme-linked immunosorbent assay

The concentration of ACTH and CORT levels in plasma are measured with commercially immunosorbent assay (ELISA) kits (56). All procedure is conducted according to the manufacturer's instructions. We first measure the absorbance of each sample at 450 nm. Then, a standard curve will be drawn, with the absorbance as the ordinate and the corresponding standard concentration as the abscissa. A regression equation from the standard curve will be used to calculate the concentration of CORT and ACTH in each sample.

#### Functional-MRI

Each subject underwent an fMRI scanner with a head orthogonal coil for fMRI data acquisition before and after acupuncture treatment. Before the scanning, participants should adjust the environment on the bed for at least 5 min to help calm down. The subject must hold still and not think systematically or fall asleep. To keep away from the scanner noise and optical disturbance, eyeshades and earplugs are put on. To keep the head from moving, foam pads are used. The scan will be performed in the following order. The position of the image involving the entire brain is structurally acquired by resting-state blood oxygenation level-dependent (BOLD) imaging and echo-planar imaging sequence (57). Then, a three-dimensional (3D) structure is included in the brain volume imaging sequences by capturing a high-resolution image of the entire brain scan (58). The parameters of each sequence scan are listed in Table 3.

**Table 3:** Scanning parameters.

**Sequence**	**TE (ms)**	**TR** **(ms)**	**Depth (mm)**	**Interval** **(mm)**	**Vision (mm^2^)**	**Matrix**
Bold	30	2000	4	0	240 × 240	64 × 64
3D-BRAVO	3.1	8.1	1	0	256 × 256	256 × 256

TE, time of echo; TR, time of repetition; BOLD, blood oxygenation level-dependent; 3D-BRAVO, three-dimensional brain volume imaging.

### Incidence of adverse events

Although acupuncture is a relatively safe treatment with a low risk of adverse events (AEs), at each acupuncture treatment, study acupuncturists will enquire about AEs. While patients receive acupuncture, AEs still may occur, such as pain, bleeding, fainting, infection, and hematoma. The rate of incidence will be carefully recorded in the case report forms, and the causality with acupuncture therapy will be analyzed.

### Data collection and management

Case report forms (CRFs) are designed to make data entry and export more convenient (55). Evaluators will record the detailed personal information and classify the research data of the subject in the CRFs. After the observed recourse end, CRFs will be sorted out in time and imported into the electronic database. To ensure the security and accuracy of databases, the completed paper CRFs will be collected into locked cabinets uniformly. In addition, the electronic database is managed by a third-party person who does not involve in the research process, so the researcher cannot modify the data content. Participants will be identified by a code and their personal information will be hidden and kept strictly confidential. The Data Monitoring Committee of the Rehabilitation Center of the First Affiliated Hospital of Guangzhou University of TCM aims to monitor the trial progress regularly, make sure the safety of the trial, and verify the completeness of the CRFs.

### Statistical analysis

#### Clinical data analysis

Third-party statisticians who do not involve in the whole assignment process and implementation of the trial will be invited to conduct statistical analysis. The data will be analyzed using SPSS 26.0 software (SPSS, Inc., Chicago, IL, USA) according to the intention-to-treat principle. The multiple imputation-expectation maximization algorithms are conducted to impute missing data. Continuous variables are summarized as mean ± standard deviation (SD) and tested using a *t*-test or Wilcoxon rank-sum test analysis. Categorical variables are reported in percentage (%) and compared using Pearson's *X*^2^ test or Fisher's exact test. The groups' comparisons are analyzed by *t*-test (independent *t*-test for comparison between the groups; paired *t-*test for comparison within the same group). The non-parametric Wilcoxon rank-sum test or the Mann-Whitney U test will be conducted for the measurement data without a normal distribution or homogeneous variance. All bilateral hypothesis tests with a significance level <0.05 will be considered statistically significant.

#### MRI data analysis

The DPARSF software platform (DPARSF, Data Processing Assistant for rs-fMRI, GNU GENERAL PUBLIC LICENSE, Beijing, China) is used to preprocess the MRI data, based on the Resting-State fMRI Data Analysis Toolkit (REST; http://www.restfmri.net) and the statistical parametric mapping (SPM8; http://www.fil.ion.ucl.ac.uk/spm). Then, the rs-fMRI data corrections are performed on the images such as slice timing and realignment for intra-volume acquisition delay. To minimize the influence of head movement, subject with head motion (maximum displacement > 2.5 mm or angle movement > 2°) during the whole fMRI scan will be excluded. Stabilization of the magnetization and adaptation of the participants to the fMRI environment will take time. For each time series, the first 10 time points will be deleted to exclude non-neuronal BOLD noise signals (59). In spatial normalization, the whole brain template of the Montreal Neurological Institute (MNI) standard space is normalized in all data spaces (isotropic voxel size = 3 × 3 × 3 mm) (60). By calculating Kendall's coefficient of concordance (KCC), a voxel-by-voxel basis of a given voxel time series with its neighboring 26 voxels is generated to obtain a separate ReHo map. Finally, a spatially smoothed function with 4 × 4 × 4 mm (full width at half maximum) Gaussian kernel is applied to reduce the noise and residual in the gyrus anatomy. The statistical analysis tool of REST is performed for independent *t*-tests to identify the ReHo patterns of the three groups (61). Besides, a paired *t*-test is applied to compare the changes in the brain before and after the treatment in each group. Covariates, such as age and education, are considered by the Pearson correlation coefficient to analyze the relationship between the improvement scores of the correlation scales and the brain regions of fMRI image data.

### Quality control

To improve quality control during the trial process, several measures will be taken. (1) Before clinical research, we will conduct a unified training for all researchers about the trial protocol, operating standard procedures, and personnel deployment. (2) To ensure the feasibility of the research operation, all the acupuncturists involved have obtained the qualification certificate for more than 5 years, and have grasped the operating specifications of the new needle equipment. (3) Instruments, equipment, and reagents to be used have strict quality standards to ensure that they work under normal conditions. (4) To ensure the reliability of the research conclusions, information feedback is carried out at the beginning and mid-term of the project, and we will solve various problems in the course of the project research promptly.

## Discussion

The individual's social role is always changed as a result of sudden neurological impairment after stroke, which requires a psychological transition and adjustment to a new definition of self (62–65). The self-acceptance process causes a high incidence of anxiety. The incidence of anxiety did not decrease significantly over time during 24 months after stroke (66). During COVID-19, PSA showed a noticeable peak (9). It has been pointed out by two comprehensive reviews that loss, social isolation, uncertainty, and physical dependence as factors that might contribute to negative psychological outcomes (67). Emotional resistance and self-acceptance are associated with poorer recovery outcomes and reduced life engagement. Since the long-term anxiety exerts a negative effect on the quality of life for the stroke survivors, which has become a worldwide phenomenon (68), it deserves more attention than it has so far received.

There has been a large number of studies showing that acupuncture is effective in the treatment of stroke and its complications. Acupuncture interventions are various, and a unified acupuncture protocol is still on the way (69). Jin's three-needle therapy (JTNT), invented by Professor Jin Rui, is famous for using three silver needles to cure diseases. JTNT is representative and has been widely used in public hospitals around Southern China. Its real clinical efficacy is worthy of study (70). Despite the high number of clinical trials about stroke, the focus of most existing research is limited to motor symptoms rather than complications after stroke (20). Besides, there is few sham control to observe the real effect of acupuncture. Investigators focus on efficacy rather than exploring pathogenesis. Given this, this study selected the sham needle equipment invented by members of our research team as the control interventions. We formulated a standard operating procedure protocol to further expound the mechanism of acupuncture through ELISA and rs-fMRI.

The clinical effect of JTNT on both motor and anxiety symptoms for PSA has been verified in the previous clinical trial carried out in the First Affiliated Hospital of Guangzhou University of TCM. To further explore the mechanism of JTNT, we completed the first draft of this research protocol. After discussion and revision of the draft by the hospital ethics committee, the final protocol was unanimously approved.

This trial attempted to assess possible mechanisms of JTNT for PSA by the HPA axis. To dynamically observe the therapeutic effect of acupuncture on PSA, we detected the key factors of the HPA axis–the changes in ACTH and CORT before and after treatment. The action mechanism of JTNT is investigated by functional magnetic resonance imaging (fMRI) to observe whether JTNT can relieve anxiety symptoms by activating the core brain regions associated with emotion regulation.

However, this study still has limitations. First, this is a single-center study, because of the COVID-19 outbreak, we cannot conduct trials in multiple centers. Secondly, due to the use of a new type of acupuncture equipment in this study, it is necessary to explain to the patient well in exchange for greater compliance. In addition, we are still working hard to standardize the process of this study to provide high-quality medical evidence for JTNT so that it can be used as an optimized plan for PSA supplementation therapy.

## Ethics statement

The studies involving human participants were reviewed and approved by Ethics Committee of the First Affiliated Hospital of Guangzhou University of Traditional Chinese Medicine. The patients/participants provided their written informed consent to participate in this study. Written informed consent was obtained from the individual(s) for the publication of any potentially identifiable images or data included in this article.

## Author contributions

Study design: ML. Study conduct: ML and YW. Drafting manuscript and approving the final version of the manuscript: all authors.

## Funding

This work was supported by the National Natural Science Foundation of China (81903971) and the Standardized Application and Clinical Evaluation of Jin's three-needle therapy for the treatment of stroke by the National Administration of Traditional Chinese Medicine (No. GZY-KJS-2020-072).

## Conflict of interest

The authors declare that the research was conducted in the absence of any commercial or financial relationships that could be construed as a potential conflict of interest.

## Publisher's note

All claims expressed in this article are solely those of the authors and do not necessarily represent those of their affiliated organizations, or those of the publisher, the editors and the reviewers. Any product that may be evaluated in this article, or claim that may be made by its manufacturer, is not guaranteed or endorsed by the publisher.

## References

[1] Liao B Geng L Zhang F Shu L Wei L Yeung PKK . Adipocyte fatty acid-binding protein exacerbates cerebral ischaemia injury by disrupting the blood-brain barrier. Eur Heart J. (2020) 41:3169–80. 10.1093/eurheartj/ehaa207 32350521 PMC7556749

[2] Feigin VL Nguyen G Cercy K Johnson CO Alam T Parmar PG . Global, regional, and country-specific lifetime risks of stroke, 1990 and 2016. N Engl J Med. (2018) 379:2429–37. 10.1056/NEJMoa1804492 30575491 PMC6247346

[3] Krishnamurthi RV Feigin VL Forouzanfar MH Mensah GA Connor M Bennett DA . Global and regional burden of first-ever ischaemic and haemorrhagic stroke during 1990-2010: findings from the global burden of disease study 2010. Lancet Glob Health. (2013) 1:e259–81. 10.1016/S2214-109X(13)70089-5 25104492 PMC4181351

[4] Rafsten L Meirelles C Danielsson A Sunnerhagen KS. Impaired motor function in the affected arm predicts impaired postural balance after stroke: a cross sectional study. Front Neurol. (2019) 10:912. 10.3389/fneur.2019.00912 31496989 PMC6713045

[5] Bourcier R Goyal M Liebeskind DS Muir KW Desal H Siddiqui AH . Association of time from stroke onset to groin puncture with quality of reperfusion after mechanical thrombectomy: a meta-analysis of individual patient data from 7 randomized clinical trials. JAMA Neurol. (2019) 76:405–11. 10.1001/jamaneurol.2018.4510 30667465 PMC6459219

[6] Wright F Wu S Chun HY Mead G. Factors associated with poststroke anxiety: a systematic review and meta-analysis. Stroke Res Treat. (2017) 2017:2124743. 10.1155/2017/2124743 28321357 PMC5340955

[7] Campbell BC Murray J Holmes J Astin F Greenwood D Knapp P. Frequency of anxiety after stroke: a systematic review and meta-analysis of observational studies. Int J Stroke. (2013) 8:545–59. 10.1111/j.1747-4949.2012.00906.x 23013268

[8] Rafsten L Danielsson A Sunnerhagen KS. Anxiety after stroke: a systematic review and meta-analysis. J Rehabil Med. (2018) 50:769–78. 10.2340/16501977-2384 30184240

[9] Ahmed ZM Khalil MF Kohail AM Eldesouky IF Elkady A Shuaib A. The prevalence and predictors of post-stroke depression and anxiety during COVID-19 pandemic. J Stroke Cerebrovasc Dis. (2020) 29:105315. 10.1016/j.jstrokecerebrovasdis.2020.105315 32958396 PMC7834239

[10] Tang WK Lau CG Mok V Ungvari GS Wong KS. Impact of anxiety on health-related quality of life after stroke: a cross-sectional study. Arch Phys Med Rehabil. (2013) 94:2535–41. 10.1016/j.apmr.2013.07.012 23911556

[11] Chun H Whiteley W Dennis M Mead G Carson A. Anxiety after stroke: the importance of subtyping. Stroke. (2018) 49:117–20078. 10.1161/STROKEAHA.117.020078 29437982 PMC5839706

[12] Hejazi-Shirmard M Lajevardi L Rassafiani M Taghizadeh G. The effects of anxiety and dual-task on upper limb motor control of chronic stroke survivors. Sci Rep. (2020) 10:15085. 10.1038/s41598-020-71845-7 32934249 PMC7492359

[13] Sanner BJ Casameni MT Cai C Tallavajhula S Hinojosa E Okpala MN . A retrospective study to identify novel factors associated with post-stroke anxiety. J Stroke Cerebrovasc Dis. (2020) 29:104582. 10.1016/j.jstrokecerebrovasdis.2019.104582 31859033

[14] Knapp P Campbell BC Holmes J Murray J Gillespie D Lightbody CE . Interventions for treating anxiety after stroke. Cochrane Database Syst Rev. (2017) 5:D8860. 10.1002/14651858.CD008860.pub3 28535332 PMC6481423

[15] Andrews G Hobbs MJ. Pragmatic treatment options for depression and anxiety disorders are needed. World Psychiatry. (2016) 15:241–2. 10.1002/wps.20364 27717253 PMC5032497

[16] Chang CC Chen TL Chiu HE Hu CJ Yeh CC Tsai CC . Outcomes after stroke in patients receiving adjuvant therapy with traditional Chinese medicine: a nationwide matched interventional cohort study. J Ethnopharmacol. (2016) 177:46–52. 10.1016/j.jep.2015.11.028 26593214

[17] Zhang S Wu B Liu M Li N Zeng X Liu H . Acupuncture efficacy on ischemic stroke recovery: multicenter randomized controlled trial in China. Stroke. (2015) 46:1301–6. 10.1161/STROKEAHA.114.007659 25873601

[18] Yue T Hongtao G. A meta-analysis of Jin's three-needle treatment for post-stroke hemiplegia. Shanghai J Acupunct Moxibustion. (2021) 40:1515–28. 10.13460/j.issn.1005-0957.2021.12.1515

[19] Qing Y Xiujin S Li S Wu G Yanbin H Yuncai W . A brief analysis of Jin's three-needle method on regulating the mind. New Chin Med. (2013) 45:100–1. 10.13457/j.cnki.jncm.2013.11.004

[20] Jun Y Depei L Weijing L Biqi H Qin Z Lixing Z. An analysis of the academic thought of situ ling, a famous lingnan acupuncturist. Chin J Tradit Chin Med. (2021) 36:804–6. Available online at: http://med.wanfangdata.com.cn/Paper/Detail?id=PeriodicalPaper_zgyyxb202102044&dbid=WF_QK

[21] Shuxin W Xun Z Muxi L Xiaoyan X Lixing Z. Zhuang lixing's clinical experience in treating postherpetic neuralgia with “tiaoshen acupuncture”. Zhongguo Zhen Jiu. (2019) 39:1095–8. 10.13703/j.0255-2930.2019.10.018 31621263

[22] Chang L Lixing Z Ting L Xiaoyan X. Zhuang Lixing's clinical experience in treating Parkinson's disease depression. Shizhen Tradit Chin Med. (2019) 30:981–2. 10.3969/j.issn.1008-0805.2019.04.074

[23] Petrik D Lagace DC Eisch AJ. The neurogenesis hypothesis of affective and anxiety disorders: are we mistaking the scaffolding for the building? Neuropharmacology. (2012) 62:21–34. 10.1016/j.neuropharm.2011.09.003 21945290 PMC3698048

[24] Papadopoulos AS Cleare AJ. Hypothalamic-pituitary-adrenal axis dysfunction in chronic fatigue syndrome. Nat Rev Endocrinol. (2011) 8:22–32. 10.1038/nrendo.2011.153 21946893

[25] Ramot A Jiang Z Tian JB Nahum T Kuperman Y Justice N . Hypothalamic CRFR1 is essential for HPA axis regulation following chronic stress. Nat Neurosci. (2017) 20:385–8. 10.1038/nn.4491 28135239

[26] Jacobson L. Hypothalamic-pituitary-adrenocortical axis: neuropsychiatric aspects. Compr Physiol. (2014) 4:715–38. 10.1002/cphy.c130036 24715565

[27] Mello AF Mello MF Carpenter LL Price LH. Update on stress and depression: the role of the hypothalamic-pituitary-adrenal (HPA) axis. Braz J Psychiatry. (2003) 25:231–8. 10.1590/S1516-44462003000400010 15328550 PMC4479183

[28] Bunea IM Szentágotai-Tătar A Miu AC. Early-life adversity and cortisol response to social stress: A meta-analysis. Transl Psychiatry. (2017) 7:1274. 10.1038/s41398-017-0032-3 29225338 PMC5802499

[29] Barra DLTP Plamondon H. Alterations in the corticotropin-releasing hormone (CRH) neurocircuitry: insights into post stroke functional impairments. Front Neuroendocrinol. (2016) 42:53–75. 10.1016/j.yfrne.2016.07.001 27455847

[30] Niu H Zhang Z Wang H Wang H Zhang J Li C . The impact of butylphthalide on the hypothalamus-pituitary-adrenal axis of patients suffering from cerebral infarction in the basal ganglia. Electron Physician. (2016) 8:1759–63. 10.19082/1759 26955446 PMC4768925

[31] Kelly PJ Murphy S Coveney S Purroy F Lemmens R Tsivgoulis G . Anti-inflammatory approaches to ischaemic stroke prevention. J Neurol Neurosurg Psychiatry. (2018) 89:211–8. 10.1136/jnnp-2016-314817 28935831

[32] Cai W Mueller C Shetty H Perera G Stewart R. Predictors of cerebrovascular event reoccurrence in patients with depression: a retrospective cohort study. BMJ Open. (2020) 10:e31927. 10.1136/bmjopen-2019-031927 31915162 PMC6955506

[33] Hoge EA Ivkovic A Fricchione GL. Generalized anxiety disorder: diagnosis and treatment. BMJ. (2012) 345:e7500. 10.1136/bmj.e7500 23187094

[34] Zang YF Zuo XN Milham M Hallett M. Toward a meta-analytic synthesis of the resting-state fMRI literature for clinical populations. Biomed Res Int. (2015) 2015:435265. 10.1155/2015/435265 26171391 PMC4478294

[35] Carter AR Astafiev SV Lang CE Connor LT Rengachary J Strube MJ . Resting interhemispheric functional magnetic resonance imaging connectivity predicts performance after stroke. Ann Neurol. (2010) 67:365–75. 10.1002/ana.21905 20373348 PMC2927671

[36] Gong J Wang J Qiu S Chen P Luo Z Wang J . Common and distinct patterns of intrinsic brain activity alterations in major depression and bipolar disorder: voxel-based meta-analysis. Transl Psychiatry. (2020) 10:353. 10.1038/s41398-020-01036-5 33077728 PMC7573621

[37] Kropf E Syan SK Minuzzi L Frey BN. From anatomy to function: the role of the somatosensory cortex in emotional regulation. Braz J Psychiatry. (2019) 41:261–9. 10.1590/1516-4446-2018-0183 30540029 PMC6794131

[38] Boehme S Ritter V Tefikow S Stangier U Strauss B Miltner WH . Brain activation during anticipatory anxiety in social anxiety disorder. Soc Cogn Affect Neurosci. (2014) 9:1413–8. 10.1093/scan/nst129 23938870 PMC4158379

[39] Wang S Yu R Tyszka JM Zhen S Kovach C Sun S . The human amygdala parametrically encodes the intensity of specific facial emotions and their categorical ambiguity. Nat Commun. (2017) 8:14821. 10.1038/ncomms14821 28429707 PMC5413952

[40] Etkin A Wager TD. Functional neuroimaging of anxiety: a meta-analysis of emotional processing in PTSD, social anxiety disorder, and specific phobia. Am J Psychiatry. (2007) 164:1476–88. 10.1176/appi.ajp.2007.07030504 17898336 PMC3318959

[41] Vizioli L Moeller S Dowdle L Akçakaya M De Martino F Yacoub E . Lowering the thermal noise barrier in functional brain mapping with magnetic resonance imaging. Nat Commun. (2021) 12:5181. 10.1038/s41467-021-25431-8 34462435 PMC8405721

[42] Zang Y Jiang T Lu Y He Y Tian L. Regional homogeneity approach to fMRI data analysis. Neuroimage. (2004) 22:394–400. 10.1016/j.neuroimage.2003.12.030 15110032

[43] Zuo XN Xu T Jiang L Yang Z Cao XY He Y . Toward reliable characterization of functional homogeneity in the human brain: preprocessing, scan duration, imaging resolution and computational space. Neuroimage. (2013) 65:374–86. 10.1016/j.neuroimage.2012.10.017 23085497 PMC3609711

[44] Lv Q Xu G Pan Y Liu T Liu X Miao L . Effect of acupuncture on neuroplasticity of stroke patients with motor dysfunction: a meta-analysis of fMRI studies. Neural Plast. (2021) 2021:8841720. 10.1155/2021/8841720 34188677 PMC8192216

[45] Association AP. Diagnostic and Statistical Manual, DSM-5 (2013).

[46] Liu L Chen W Zhou H Duan W Li S Huo X . Chinese stroke association guidelines for clinical management of cerebrovascular disorders: executive summary and 2019 update of clinical management of ischaemic cerebrovascular diseases. Stroke Vasc Neurol. (2020) 5:159–76. 10.1136/svn-2020-000378 32561535 PMC7337371

[47] Lim S. WHO standard acupuncture point locations. Evid Based Complement Alternat Med. (2010) 7:167–8. 10.1093/ecam/nep006 19204011 PMC2862941

[48] Zhou W Benharash P. Significance of “Deqi” response in acupuncture treatment: Myth or reality. J Acupunct Meridian Stud. (2014) 7:186–9. 10.1016/j.jams.2014.02.008 25151451

[49] Pan H Zhao Y Li J Wen Q Li N. Primary discussion of qualitative and quantitative recognition on deqi after acupuncture: a study report of West China School of Medicine. Zhongguo Zhen Jiu. (2015) 35:67–71. 10.13703/j.0255-2930.2015.01.020 25906574

[50] Si X Han S Zhang K Zhang L Sun Y Yu J . The temporal dynamics of EEG microstate reveals the neuromodulation effect of acupuncture with deqi. Front Neurosci. (2021) 15:715512. 10.3389/fnins.2021.715512 34720853 PMC8549605

[51] Thompson E. Hamilton rating scale for anxiety (HAM-A). Occup Med. (2015) 65:601. 10.1093/occmed/kqv054 26370845

[52] Ahn JK Kim Y Choi KH. The psychometric properties and clinical utility of the korean version of GAD-7 and GAD-2. Front Psychiatry. (2019) 10:127. 10.3389/fpsyt.2019.00127 30936840 PMC6431620

[53] Plummer F Manea L Trepel D McMillan D. Screening for anxiety disorders with the GAD-7 and GAD-2: a systematic review and diagnostic metaanalysis. Gen Hosp Psychiatry. (2016) 39:24–31. 10.1016/j.genhosppsych.2015.11.005 26719105

[54] Saber H Saver JL. Distributional validity and prognostic power of the national institutes of health stroke scale in US administrative claims data. JAMA Neurol. (2020) 77:606–12. 10.1001/jamaneurol.2019.5061 32065612 PMC7042858

[55] Ohura T Hase K Nakajima Y Nakayama T. Validity and reliability of a performance evaluation tool based on the modified Barthel Index for stroke patients. BMC Med Res Methodol. (2017) 17:131. 10.1186/s12874-017-0409-2 28841846 PMC6389202

[56] Ran YH Hu XX Wang YL Zhao N Zhang LM Liu HX . YL-0919, a dual 5-HT(1A) partial agonist and SSRI, produces antidepressant- and anxiolytic-like effects in rats subjected to chronic unpredictable stress. Acta Pharmacol Sin. (2018) 39:12–23. 10.1038/aps.2017.83 28858297 PMC5758671

[57] Kim SG Ogawa S. Biophysical and physiological origins of blood oxygenation level-dependent fMRI signals. J Cereb Blood Flow Metab. (2012) 32:1188–206. 10.1038/jcbfm.2012.23 22395207 PMC3390806

[58] Fuchigami T Shikauchi Y Nakae K Shikauchi M Ogawa T Ishii S. Zero-shot fMRI decoding with three-dimensional registration based on diffusion tensor imaging. Sci Rep. (2018) 8:12342. 10.1038/s41598-018-30676-3 30120378 PMC6098116

[59] Fleischmann R Decker AM Kraft A Mai K Schmidt S. Mobile electronic versus paper case report forms in clinical trials: a randomized controlled trial. BMC Med Res Methodol. (2017) 17:153. 10.1186/s12874-017-0429-y 29191176 PMC5709849

[60] Das S Glatard T Rogers C Saigle J Paiva S MacIntyre L . Cyberinfrastructure for open science at the montreal neurological institute. Front Neuroinform. (2016) 10:53. 10.3389/fninf.2016.00053 28111547 PMC5216036

[61] Chao-Gan Y Yu-Feng Z. DPARSF: a MATLAB toolbox for “pipeline” data analysis of Resting-State fMRI. Front Syst Neurosci. (2010) 4:13. 10.3389/fnsys.2010.00013 20577591 PMC2889691

[62] Harrison M Ryan T Gardiner C Jones A. Psychological and emotional needs, assessment, and support post-stroke: a multi-perspective qualitative study. Top Stroke Rehabil. (2017) 24:119–25. 10.1080/10749357.2016.1196908 27309492

[63] Crowley D Andrews L. The longitudinal relationship between acceptance and anxiety and depression in people who have had a stroke. Aging Ment Health. (2018) 22:1321–8. 10.1080/13607863.2017.1348478 28727485

[64] Wray F Clarke D. Longer-term needs of stroke survivors with communication difficulties living in the community: a systematic review and thematic synthesis of qualitative studies. BMJ Open. (2017) 7:e17944. 10.1136/bmjopen-2017-017944 28988185 PMC5640038

[65] Townend E Tinson D Kwan J Sharpe M. 'Feeling sad and useless': an investigation into personal acceptance of disability and its association with depression following stroke. Clin Rehabil. (2010) 24:555–64. 10.1177/0269215509358934 20483889

[66] Unsworth DJ Mathias JL Dorstyn DS. Preliminary screening recommendations for patients at risk of depression and/or anxiety more than 1 year poststroke. J Stroke Cerebrovasc Dis. (2019) 28:1519–28. 10.1016/j.jstrokecerebrovasdis.2019.03.014 30928216

[67] Foley EL Nicholas ML Baum CM Connor LT. Influence of environmental factors on social participation post-stroke. Behav Neurol. (2019) 2019:2606039. 10.1155/2019/2606039 30800187 PMC6360065

[68] Andersson J Stålnacke BM Sörlin A Magaard G Hu X. Long-term perceived disabilities up to 10 years after transient ischaemic attack. J Rehabil Med. (2021) 53:m167. 10.2340/16501977-2808 33656562 PMC8814880

[69] Lu L Zhang XG Zhong LL Chen ZX Li Y Zheng GQ . Acupuncture for neurogenesis in experimental is chemic stroke: a systematic review and meta-analysis. Sci Rep. (2016) 6:19521. 10.1038/srep19521 26786869 PMC4726177

[70] Qing Y Longlin L Xiujin S Fei C Yuncai W. Discuss the academic connotation of “Jin's Three Needles”. Chin Acupunct Moxibustion. (2014)34:701–4.

